# Book Review

**Published:** 1923-07

**Authors:** 


### Reviews of Books.

**Elements of Pharmacy, Materia Medica and Therapeutics.** By Sir William Whitla, M.P., M.D., Etc., 1923. Eleventh Edition, 47th Thousand. London: Baillière, Tindall and Cox.—This excellent old friend is invaluable both to the student and to the practitioner, as it contains such an extraordinary amount of information on all three subjects. Indeed, we know of no other work at once so comprehensive and so exact. It has been again revised and brought up to date, and an immense number of non-official remedies are ably described and their claims discussed. It now includes a directory of what to do in case of poisoning, where many new remedies may be found, such as adrenalin and permanganate in strychnine and cyanide cases. In the treatment of carbon monoxide poisoning we wish that it had been said that the oxygen should be given under pressure, the open method being unreliable.

**Reports of the St. Andrew's Institute for Clinical Research.** Vol. I. London: Henry Frowde.—This Institute is one of the great romances of our time. Sir James Mackenzie, after a successful life, feeling deeply the difficulties in the way of the advancement of medical learning, has settled down with a little band of helpers to think out a fresh *Novum Organon*, and to revise our logical methods for the study of disease. Like his brother Scot, who prayed, "Lord, have mercy on the poverty of our art," he recognises keenly the limitations of medicine, and in place of improved chemical or other laboratory methods he looks for progress from minute observation of clinical symptoms, especially those in the earliest stages of disease, and the continuance of this watching through the entire course of the disease. Whether or no his quest will revolutionise medicine, a perusal here of his methods of case taking shows the very great amount to be learnt from the minute study of a single clinical symptom, such as pain or exhaustion. He points out that symptoms are the reaction of the tissues to the injurious agent which causes the ill-health, and that most of them are disturbances of normal reflexes.

**A Manual of Surgical Anatomy.** By Lewis Beesly, F.R.C.S., Edin. and T. B. Johnston, M.B., Ch.B. Second Edition. Pp. xiv., 561. London: Oxford Medical Publications. 1922. Price 18s.—This edition of a text-book which has attained to considerable popularity, has been revised and brought up to date. It treats the subject from an anatomical point of view, and it must be admitted that the inclusion here and there of an occasional clinical case would have the effect of making it more readable. The student in his later years finds his days very full, and it is much to be doubted if he spends many hours in systematic reading of surgical anatomy ; he might be tempted to spend more if it were presented to him with its clinical bearing a little more strongly emphasised. We note with regret that the Basle terminology has been retained ; we were under the impression that it was as discredited by anatomists as it is ignored by clinicians. Certainly the student knows little of it, and even less of the classics. An examiner seeking information about " Dislocations of the Talus " would not generally receive a ready reply. War lessons receive attention especially in the matter of nerve injuries, but we should have liked to see the question of infection of the knee-joint treated differently, in the light of war experience. One thing the war taught us was that to put drainage tubes into an infected knee-joint was generally to condemn the patient to the loss of his limb, if not his life ; yet here we find precise directions for inserting numerous tubes into the joint. These are small blemishes where generally there is room for nothing but praise. Certainly the candidate for even the highest examinations can face his ordeal with confidence if he has a good working knowledge of the subject as presented to him here. The excellent illustrations, many of them in colour, deserve a special word of commendation.

**De Arte Phisicali et De Cirurgia of Master John Arderne, Surgeon, of Newark, dated 1412.** Translated by Sir D'Arcy Power, K.B.E., M.B. Oxon., F.R.C.S. Pp. xii., 60. London : John Bale, Sons & Danielsson Ltd. 1922. Price 10s. 6d. net.—We owe this unique and beautiful volume to the Wellcome Museum, which came into the possession of a photographic copy of a MSS. of this treatise existing in the Royal Library at Stockholm. The treatise appears to be an epitome of one or more of Arderne's works compiled in 1412, some years after his death, for everyday use. It consists chiefly of directions for treatment and prescriptions for various conditions from flatulence to pleurisy, periostitis, and calculi. Brief instructions for the management of different fætal presentations in labour show when turning is needed and how delivery can be effected. The translation is wonderfully clear, and Sir D'Arcy Power's knowledge of Arderne has enabled him to supply missing words and elucidate doubtful passages from other writings of his. Twelve sections of the photographed MSS. are here reproduced with the quaint original illustrations.

These beautiful plates give some idea of the heavy task of Mr. Eric Millar in transcribing the text. The treatise itself does not, however, do justice to Arderne, omitting as it does so many of his *obiter dicta*, and showing little of his real surgical skill, while it embodies many popular superstitions of the time which could be gleaned from his writings. It is interesting to find among the strange remedies proposed a simple hot brick recommended for stomach ache, and to be told that wine is hurtful in every nerve complaint.

**Greffes Testiculaires.** Par Le Docteur SERGE VORONOFF. Pp. 83. Paris: Libraire Gaston Doiu. 1923.—This is an extremely interesting resumé of the work done by Dr. Voronoff on the effects of testicular deprivation and implantation in animals, founded on 120 experiments. He has justified these in, apparently, obtaining rejuvenating effects in the senile man by the implantation of testicular fragments from the anthropoid apes. The communication is verified by the results of retardation of sexual characteristics, by the development of characters found in the opposite sex, by regeneration of the senile animal, and by obtaining in man also apparent rejuvenation. The results shown photographically and the microscopic findings bear out his contention that the testicular hormone survives in the transplant and influences the host, and that the undeveloped testicle even tends towards full development. The book bears the stamp of honest investigation and is well worth reading.

**Hewitt's "Anæsthetics and their Administration."** Edited by HENRY ROBINSON, M.A., M.D., B.Ch. Fifth edition. Pp. xiii., 576. London: Oxford Medical Publications. 1922. Price 30s. net.—Dr. Robinson has successfully performed the difficult task of re-editing the popular textbook on anæsthetics written by the late Sir Frederic Hewitt, and he has obviously spared himself no trouble in seeing that every section of the work is abreast of the times. We are sorry that the Index of Authors has been omitted. The new account of the Physiology of Anæsthesia written by Professor Clark will be found full of useful information described in very lucid language. The better-known theories of the way in which these drugs act are given briefly with short comments as to their feasibility. This section includes some very practical references to the influence of anæsthetics in the production and prevention of shock. Dr. Robinson is a strong advocate of nitrous-oxide and oxygen, also of spinal anæsthesia, and these two methods have received careful treatment which will be appreciated by practitioners and students alike. By so able a revision the continued popularity of this old favourite has been ensured.

**Medical Report of the Tainan Hospital, English Presbyterian Mission, Formosa, 1920-1922.** (For distribution among the Medical Profession only.) By JAMES L. MAXWELL, M.D., B.S. Lond. Pp. 26.—Two European medical men, working in a hospital of 150 beds, have in less than three years treated over 6,500 in-patients and over 25,000 out-patients. These are the bare facts which this short report sets forth, with details which go to show that the experience to be gained in this and similar hospitals is of the first quality. This argument, together with the urgent need for workers, may bring young graduates who are keen on their profession to consider whether the career of a medical missionary may not have its attractions. This pamphlet, which includes an extremely interesting tabular summary of the surgical work done during the period under review, may sound the " call " to some of them.

**Selected passages from " De l'Auscultation Médiate."** By R. THÉOPHILE H. LAENNEC, with a biography by Sir WILLIAM HALE-WHITE, K.B.E., M.D. Pp. x., 193. *Medical Classics Series.* London: John Bale, Sons & Danielsson. 1923. Price 12s. 6d. net.—It is a good thing to be called upon from time to time to remember famous men and our fathers that begat us. The *Medical Classics Series* comes most opportunely, at a time when physical examination runs some risk of being overshadowed by laboratory examination and " mail-order " diagnostics, to remind us that the men of past times made discoveries and progress no less striking than our own. Sir William Hale-White in his biographical preface to this translation gives three reasons for the immediate success of Laennec's book on Auscultation. Firstly, because it contains perfect, precise and original descriptions of clinical symptoms and post-mortem appearances. Secondly, on account of the description of auscultation with a stethoscope, which he invented on the spur of a singularly happy moment. Thirdly, by reason of the beauty of language and the economical precision of words which makes Laennec a pleasure to read. The opening words of his first chapter (on voice sounds) are strikingly reminiscent of Auenbrugger. Laennec begins, " When a healthy man speaks or sings his voice reverberates within his chest " ; Auenbrugger had written nearly sixty years before : " The chest of a healthy man resounds when it is struck." From these two observations two men made out the whole art of physical examination of the chest, Laennec completing the great work that Auenbrugger had begun, with frequent acknowledgment of his debt to that master and his enthusiastic disciple Corvisart. Many books have been published for the benefit of students starting in the wards and intended to explain in simple language the difficult practices of percussion and auscultation. But for simplicity Laennec stands above them all. Take, for example, the *crepitant râle,* which he says is the pathognomonic sign of the first stage of peri-pneumonia, "a kind of crepitation comparable to the crackling sound produced by salt heated on a frying-pan." We may well apply to Laennec his own remarks on Bayle with which he concluded his chapter on Metallic Tinkling: "It is given to no man to see everything. But what he did see, he saw superlatively well, and there are few books in which there is less that requires deleting than in his." To medical students, who may come under reproof for idle and depraved recreations, we offer as some consolation that Laennec's devotion in his youth to music, dancing and rambles in the country worried his uncle (who was one of his teachers in hospital) not a little; especially music, which he loved best.

**Suggestion and Common Sense.** By R. Allan Bennett, M.D. Pp. 105. Bristol: John Wright & Sons Ltd. 1922. Price 6s. net.—The author, in his preface, frankly admits that he has always "shunned psychology and clung to that which is good," whatever the latter phrase may mean. The reader may be excused, therefore, if he finds difficulty in accepting the author's psychological concepts as set out in the first two chapters. In his first chapter, on "Psychology and Organic Life," he misuses the term consciousness by broadening the meaning out of all recognition. When he wishes to use the word in the accepted sense he finds himself obliged to introduce a qualification, and speak of "conventional consciousness." By what right is the physiological response of tissues or isolated cells to be termed "conscious" or "intellectual"? Can we call a phagocyte intellectual? If the author had described the phenomena of adaptation in organic life in terms of specific response or behaviour the first chapter would be full of interest and free from objection. Consciousness of the individual cell is unthinkable. The writer evidently wishes to give some description of his experiences and successes in the field of psychotherapy. In so far as he sticks to the narration of experience he is interesting, though the subjects have been covered many times before and more scientifically. The book is pleasant reading, if not profound. One is left with the impression that in the writer's estimate common sense belongs to a higher category of thought than scientific thinking.

**The New Physiology in Surgical and General Practice.** By A. Rendle Short, M.D., B.S., M.C.S. Fifth edition. Pp. xi., 330. Bristol: John Wright & Sons Ltd. 1922. Price 9s. 6d. net.—Since 1911, when this book was originally published, several editions have appeared, so that but little of the contents of the first is to be found in the fifth. But this work preserves its essential features, and presents a number of separate essays on those portions of physiology which are of special interest to physicians, surgeons and general practitioners. It also possesses an interest for everyone who, like the author, believes that an intimate relationship should exist between physiology and medicine. This view is not universally held, since the teaching of physiology is often controlled by those who not only have no medical qualification, but are quite unsympathetic to the needs of medical students or of medical men. This work no longer limits its interest to surgical problems and to the common everyday aspects of disease. This is easily realised when such subjects as the physiology of muscular exercise and the functions of the kidney are included. These are admirably discussed, while the diseases dependent on deficiences in foods are considered at some length. An excellent chapter on the heart, occupying some forty pages of the book, is contributed by Dr. C. E. K. Herapath. Difficult as it is to adequately show how the research work of laboratories has a direct bearing on practice, it is even harder to impress this truth. Perusal of this book, which succeeds in accomplishing what is certainly a matter of some difficulty, will show how physiological work, or rather physiological knowledge, may be considered as a science which has a direct application to medicine. Apart from this the work is full of interest, more so according to one reviewer than a novel.

**The Surgical Diseases of Children.** By F. C. Pybus, M.S., F.R.C.S. Pp. xviii., 408. London: H. K. Lewis & Co. Ltd. 1922.—This book meets a distinct want, as no volume of the kind has of late been published, and students are compelled to hunt through manuals of general and orthopædic surgery for any account of the surgical ailments peculiar to children. In parts the book is somewhat sketchy, and the details of operative treatment might be amplified with advantage, but the commoner conditions, with their treatment, are very clearly and concisely described. Attention may well be drawn to the author's warning against the over-preparation of children for operation. This is a very common and serious fault, and preliminary purgation and starvation are still too often employed, with disastrous consequences. In acute osteitis Mr. Pybus is able to show most excellent results from complete and partial diaphysectomy. The operation deserves to be more widely practised, as it is undoubtedly an immense improvement upon the less radical methods still commonly employed in the treatment of this disease. It may interest Bristol readers to know that the late Mr. Stack performed a complete subperiosteal removal of the shaft of the radius as long ago as 1908 with a perfect result both as regards function and bone regeneration. Mr. Pybus's book does not pretend to be an exhaustive manual of the surgery of children, but is based almost entirely upon his own personal experience and practice, and as such it may confidently be recommended. The senior student desirous of obtaining some special knowledge of a very important branch of surgery will find this very readable and up-to-date little book particularly valuable.

**The Sympathetic Nervous System in Disease.** By W. Langdon Brown, M.D., F.R.C.P. Second Edition. Pp. xi., 161. London: Oxford Medical Publications. 1923. Price 10s. 6d. net.—The progress made during recent years in our knowledge of this subject has necessitated the publication of a second edition within three years of the first. No higher praise can be afforded to it than to say that it retains the charm of the first edition, losing nothing in its masterly condensation of a large mass of nebulous data into a form that can be clearly surveyed by the least expert of students. It is a book that ought to be read by every practitioner of medicine, and for this reason we wish the cost of the book were not so high in proportion to its bulk.

**A Synopsis of Surgery.** By Ernest W. Hey Groves, M.D., M.S., F.R.C.S. Sixth Edition. Pp. viii., 621. Bristol: John Wright & Sons Ltd. 1922. Price 17s. 6d.—This book was bound to succeed. It is so full and yet so handy; it gives such a wealth of information at a glance, and is written with such discretion, that it is no matter for surprise that it has run to six editions. As might be expected from the distinguished author, the chapters on fractures and deformities have been brought well up to date and illustrated. Perhaps it is too much to expect that in a single small volume everything in surgery should be thoroughly modernised, but we think in the next edition several not very rare conditions might deserve a word, *e.g.* idiopathic renal hæmaturia, B. coli infections of the prostate, sacroiliac strain, local tetanus, mobile cæcum mimicking appendicitis, etc. It is not mentioned that a valuable sign of gastric carcinoma is a filling defect in the X-rays, and the value of X-ray treatment for lymphadenoma might be mentioned. Some statements would scarcely receive wide acceptance to-day; for instance, it is stated that the normal stomach empties in two hours, and that $MgSO_4$ is "of great value" in the treatment of tetanus. In the treatment of trigeminal neuralgia removal of the ganglion is spoken of as the method of choice in all severe cases, and division of the sensory root, which many would prefer, is said to be done through the *posterior* cranial fossa, which is not the best modern method in our judgment. However, surgery is not a finished science, and no one will ever write a book that will command universal acceptance for every sentence. The book is beautifully turned out, as befits the house that prints it, which is a credit to our town.

**Tumours Complicating Pregnancy, Labour, and the Puerperium.** By Herbert R. Spencer, M.D., B.S. Pp. 78. London: Harrison & Sons. Price 5s. net.—This book is a reprint of the author's Lettsomian lectures; it is unnecessary to say that the general presentation of the subject and the accuracy of the clinical observations recorded in the subject matter of the lectures are all that might be expected from the author. The lectures form a most valuable contribution to the literature on the subject: the clinical material is ample, its analysis masterly, and the conclusions drawn therefrom based on sound logical reasoning. Dealing with the cases in detail, the author shows that as regards fibroids a quite definite proportion of cases in which these tumours complicate pregnancy require operative treatment, as operative emergencies necessitated by degenerative processes occurring in the tumour, and that of these, unless operation is unduly delayed, a large proportion can be dealt with by myomectomy without interrupting the pregnancy. The author's findings are in accord with the experience of most of those who have found it necessary to deal with cases of the kind. Of the cases dealt with by Cæsarean section it would perhaps have been better to exclude, or to place in a separate table, those in which the operation was necessitated by contracted pelvis and not primarily by the fibroid. This, however, does not prevent sound deductions as to the necessary indications for operations being drawn from the cases quoted. As regards ovarian tumours, the presentation of the cases is most complete and the deductions drawn are very sound. The treatment is worth summarising as follows: 1. During the first half of pregnancy ovarian tumours (with a few exceptions) should be removed wherever their situation and whatever their size. 2. During the second half of pregnancy: (*a*) All large tumours and all others showing signs of pathological changes should be removed; (*b*) small *abdominal* tumours not producing symptoms may be left if under constant observation. 3. During labour immediate ovariotomy for *all large* tumours. Small tumours at end of first stage or immediately after delivery. Cæsarean section is necessary in rare cases of adherent or solid tumours in pelvis, but no such measures as induction of labour, forceps, version or tapping to relieve dystocia should be considered.

**Lawson Tait: His Life and Work.** By W. J. Stewart McKay, M.B. Pp. xii., 579, London: Baillière, Tindall & Cox. 1922. Price 25s. net.—This work gives a most interesting account of the life and work of one who was, by common consent, regarded during his life as one of the pioneers of Surgery. It will be of interest to those who knew him personally, but probably even more interesting to those who knew of him only as a contemporary worker. It is a record of a man possessed of an enormous capacity for overcoming difficulties, of boundless energy and unflagging industry; who was possessed of exceptional clinical acumen and manual dexterity, which made him an outstanding figure in a generation which contained many giants. The soundness of his work is attested by the fact that most of his methods have been preserved by his successors, thus proving that they were based on sound surgical reasoning. The cases cited in connection with controversies which arose over various procedures which he advocated make particularly good reading. Space does not allow a detailed account of these, but it must be noted with regard to Tait's operation and methods in ectopic gestation that although he discarded, and rightly, the vaginal route in dealing with this particular complication, his assistant and successor, Professor Taylor of Birmingham, made use of it not infrequently in our experience, as a method of clearing up the diagnosis in doubtful cases, when for any reason it did not seem advisable to perform an exploratory laparotomy for that purpose. A certain degree of intolerance, perhaps most marked in his attitude towards Lister and his methods, is to be noticed, as also a certain capacity for special pleading which at times approaches the boundary of strict scientific truth. The latter is demonstrated in his controversial speeches and writing. In spite of this, the author presents the picture of a man of enormous capacity, both intellectual and physical, of great originality and strong personality. There is little doubt that a certain harshness in dealing with opponents, a degree of intolerance of the opinions of others, and his special pleading were due to his early difficulties and to the opposition to which, he, like all other pioneers, was exposed. The methods of some of his opponents, as described by the author, were the reverse of creditable, and, as we know, jealousy was responsible for a good deal. The author depicts Tait in his private life as he was—somewhat of a *viveur,* a sportsman, a genial companion, a firm friend, a connoisseur of art and literature and, in fact, a man *teres atque rotundus*.

**Venereal Disease in the American Expeditionary Forces.** By George Walker, M.D., late Colonel, Medical Corps, U.S.A. Pp. xxiii., 237. Baltimore; Medical Standard Book Co. 1922. —This book shows the methods which really proved effective in the control of Venereal Disease in France. The author states, " After a close study of conditions in France, it became evident that in order to limit very materally venereal infection, our chief reliance would have to be placed on prophylaxis." The most stringent measures were adopted to prevent the incidence of the disease. General Pershing commenced the campaign by penalising every patient. This was soon followed by an order by which failure to apply prophylaxis was also punished. Owing to the great loss of time incurred by the British and French Armies, because of their system of treating Venereal Disease in base hospitals, it was decided to retain venereal patients with their own units and treat them at the regimental infirmary, except when complications existed in which case the men were sent to special hospitals. When Divisions went into line the Venereal Disease victims were detained in the area of the Divisional operations. It was found that 71 per cent. of all American soldiers in France had sex relations during the stay in that country. Before orders were enforced for compulsory prophylaxis, venereal infection amongst the first troops in France was found to be 240 per thousand per year among the whites, and 625 per thousand per year amongst the coloured. Compulsory prophylaxis soon reduced the rate to almost zero per thousand among the whites, and 20 per thousand among the coloured. A record of 242,000 exposures showed only 1.3 per cent of failure. All leave areas, Red Cross huts, and even leave trains provided stations with skilled attendants and medical officers in charge. Comparative incidence of Venereal Disease showed Gonorrhœa, 52 per cent ; Chancroid, 40 per cent ; Syphilis, 8 per cent. An interesting fact was that 42 per cent. of all sores proved by dark field illumination to be syphilitic. The book is pleasantly written, disclosing faithful and painstaking compilation from a multitude of sources, and will well repay study.

